# Supplementary material for: The transcription factor ERG regulates a low shear stress-induced anti-thrombotic pathway in the microvasculature
Source: Nat Commun. 2019 Nov 1;10:5014. doi: 10.1038/s41467-019-12897-w (PMC6825134; doi:10.1038/s41467-019-12897-w)
Supplement: Supplementary file 1 — Supplementary Information [file 41467_2019_12897_MOESM1_ESM.pdf]

Supplementary Information

The transcription factor ERG regulates a low shear stress-induced anti-thrombotic pathway in the microvasculature

Peghaire et al.

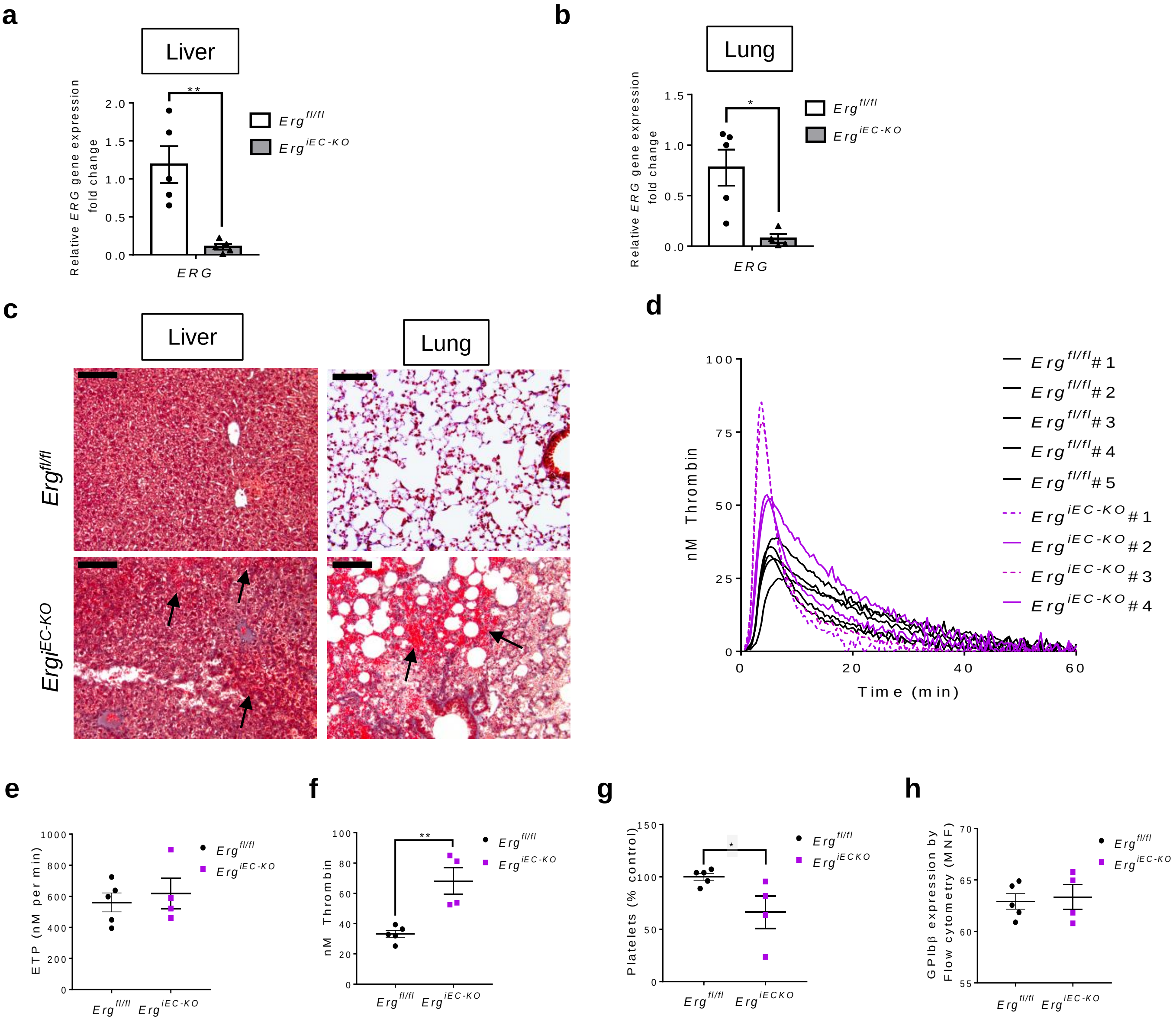

Supplementary Figure 1: Deletion of ERG in *vivo* is associated with impairment of coagulation and bleeding. (**a-b**) qPCR analysis of *ERG* gene expression in whole liver (**a**) and lung (**b**) lysates from adult control ($Erg^{fl/fl}$) and ERG-deficient ($Erg^{iEC\text{-}KO}$) mice (n=5 per genotype). Data were normalized to 18S. (**c**) Representative image of Masson's Trichrome staining performed on liver and lung sections from $Erg^{fl/fl}$ and $Erg^{iEC\text{-}KO}$ mice, 45 days after tamoxifen injection, and showing the presence of extravasated red blood cells (black arrows). Scale bar 100 µm. (**d-f**) Thrombin generation using CAT assay was performed on plasma from $Erg^{iEC\text{-}KO}$ and $Erg^{fl/fl}$ mice 45 days post tamoxifen injection. (**d**) Thrombin generation was determined from the accumulation of fluorescent product over time and calculated relative to a thrombin calibrator. (**e**) Endogenous thrombin potential (ETP) (nM per min) and (**f**) peak height (nM) of plasma from $Erg^{iEC\text{-}KO}$ and $Erg^{fl/fl}$ mice. Both parameters were calculated and plotted as mean per individual mouse (n=4-5 per genotype). (**g**) Platelet counts (Platelets x10^3 per µl expressed as % of control mice) were determined on plasma from *ErgiEC-KO* and *Ergfl/fl* mice, 45 days post tamoxifen (n=4-5 per genotype). (**h**) Expression of the platelet marker GP1bβ in $Erg^{fl/fl}$ and $Erg^{iEC\text{-}KO}$ mice was measured by flow cytometry (n=4-5 per genotype). All graphical data (except data presented in **d**) are mean ±s.e.m., *P<0.05, **P<0.01, Student's t-test. Source data are provided as a Source Data file.

a

Phenotype 30 days post tamoxifen

Liver clots	Liver hemorrhages	Liver C+H	Lung hemorrhages	Platelet counts	Fibrinogen	D-dimer	TAT
77.8% (7/9)	66.7% (6/9)	55.6% (5/9)	55.9% (5/9)	Decreased: 37.5% (3/8)	Decreased: 50% (4/8)	Increased: 87.5% (7/8)	Increased: 62.5% (6/8)

b

Phenotype 45 days post tamoxifen

Liver clots	Liver hemorrhages	Liver C+H	Lung hemorrhages	Platelet counts	D-dimer	Thrombin Generation
100% (4/4)	75% (3/4)	75% (3/4)	50% (2/4)	Decreased: 75% (3/4)	Increased: 50% (2/4)	Increased: 100% (4/4)

Supplementary Figure 2: Variable penetrance of the phenotype and progression of the disease in adult ERG-deficient mice (**a**) 30 and (**b**) 45 days post tamoxifen injection. The values represent the percentage of *Erg*$^{iEC\text{-}KO}$ mice showing the depicted phenotype. Numbers in brackets are: the number of *Erg*$^{iEC\text{-}KO}$ mice with the specified phenotype and the total number of *Erg*$^{iEC\text{-}KO}$ mice analysed for the study. C+H: presence of clots and hemorrhages in liver

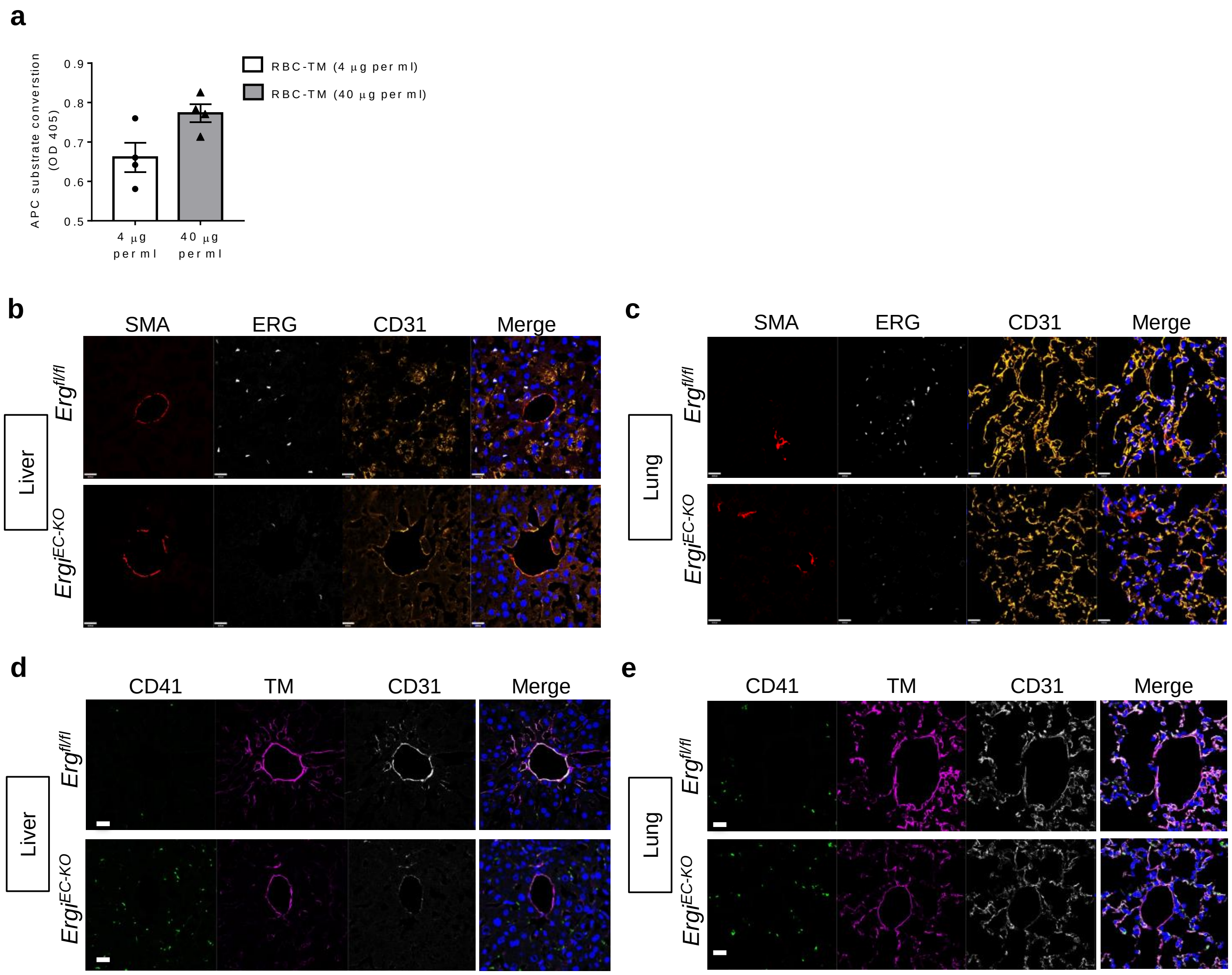

Supplementary Figure 3: Controls for *in vivo* rescue experiment using RBC-TM. (**a**) APC generation assay *in vitro*. Free RBC-TM (4 or 40 µg per ml) was incubated with 5 nM bovine thrombin and 300 nM human protein C for 20 minutes at room temperature. Thrombin was quenched with hirudin (50 U per mL), and APC was measured using Spectrozyme (OD at 405nm). Samples were run in quadruplicates. Graphical data are mean ±s.e.m., presented as maximum signal reached at 20 min. (**b-c**) Confirmation of ERG deletion was assessed by immunofluorescence in (**b**) liver and (**c**) lung sections from adult *Erg*$^{fl/fl}$ and *Erg*$^{iEC\text{-}KO}$ mice 25 days after tamoxifen injection (6 hours after RBC-TM injection). ERG is shown in grey; sections are co-stained for CD31 (yellow) and SMA (Smooth muscle actin) (red) to visualize blood vessels and nuclei are identified by DAPI (blue). Scale bar 50 µm. (**d-e**) Confirmation of TM decreased expression was assessed by immunofluorescence in (**d**) liver and (**e**) lung sections from adult *Erg*$^{fl/fl}$ and *Erg*$^{iEC\text{-}KO}$ mice 25 days after tamoxifen injection (6 hours after RBC-TM injection). TM is shown in magenta; sections are co-stained for CD31 (grey) to visualize blood vessels and CD41 (green) to visualize platelets; nuclei are identified by DAPI (blue). Scale bar 50 µm. Source data are provided as a Source Data file.

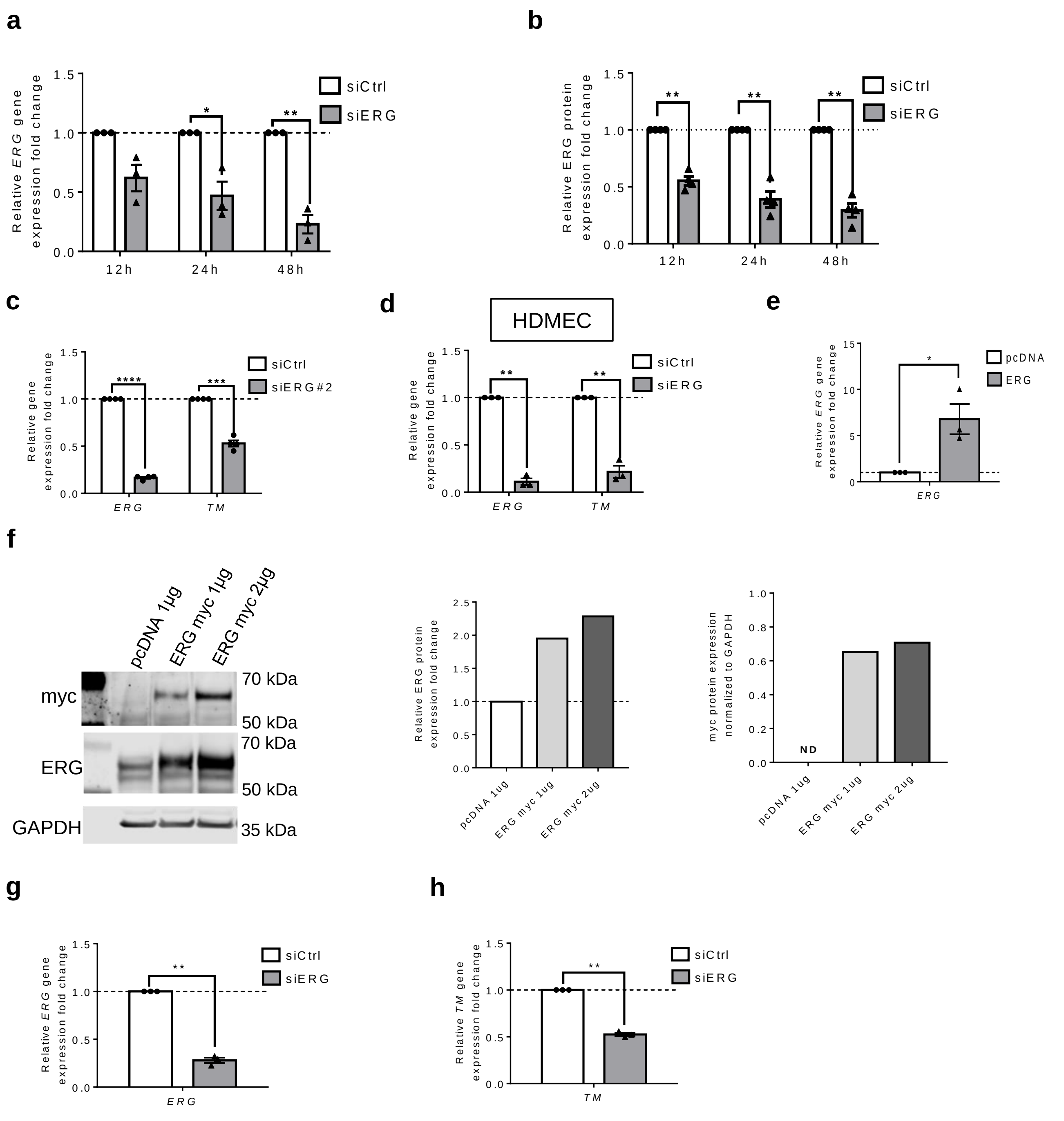

Supplementary Figure 4: ERG controls the expression of thrombomodulin in macro- and micro-vascular endothelial cells in static conditions. (**a**) qPCR (n=3 independent experiments) and (**b**) immunoblotting (n=4 independent experiments) analysis of ERG expression in control (siCtrl) and ERG-deficient (siERG) HUVEC (macrovascular EC) after 12, 24 and 48 hours siRNA treatment. (**c**) qPCR analysis of *ERG* and *TM* gene expression following transfection of HUVEC with a second siRNA targeting exon7 of ERG (siERG#2) (n=4). (**d**) qPCR analysis of *ERG* and *TM* expression in control (siCtrl) and ERG-deficient (siERG) HDMEC (microvascular EC) after 48 hours siRNA treatment (n=3 independent experiments). (**e**) *ERG* mRNA expression (n=3 independent experiment) and (**f**) representative immunoblotting and quantification of ERG and myc in HUVEC transfected with control pcDNA or ERG expression plasmid (myc tagged, noted ERG). (**g**) *ERG* and (**h**) *TM* mRNA levels in HUVEC treated with siCtrl or siERG for 48 hours and used for APC assays (n=3 independent experiments). All graphical data (except data presented in **f**) are mean ±s.e.m., *P < 0.05, **P < 0.01, ***P < 0.001, ****P < 0.0001, Student's t-test. Source data are provided as a Source Data file.

a

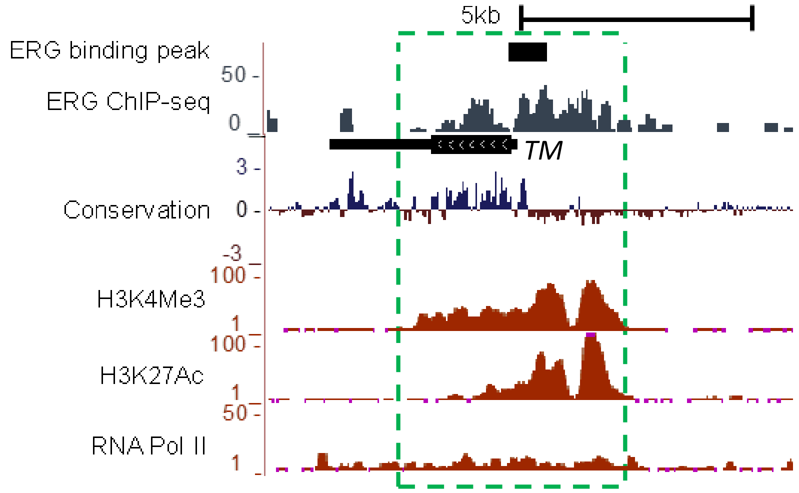

b

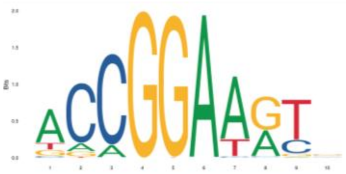

c

ERG peak sequence on the TM promoter(-559bp;+215bp)

```
>hg19_dna range=chr20:23030101-23030909 5'pad=0 3'pad=0
strand=+ repeat Masking=none
    CCGGCCAGGGCCAGCGCGCCAAGGACCAGGACCCCAAGCATGTTACCCAG
51  GCGCGCCGCGTGCAGGCGCCGGGGAAAGCGCGGGCACTGCGACAGGGCCG
    TGCCGGAGCAGAGGGGCACAGGACGCCGATGGCGACAGCCTCTCCTGTCC
151 GTCCCAGCCCAGACACTTCTTGCCGCTGCGCGCAGCCCCTGCGAGGCAGC
    CTCTGACATGCGGATCGGCCAGGGCTCGAGTTTATAAGTGCCCGGCCCTC
251 CCTCCCTGGACGTTCGGGAAAAGGAAGGAAGTGCCTGGTGGGAAGGGCTG
    ATGCCGCATACTCGGATTGCTGGGTTCTCTGGCCGCCCTTGCGCCCGCCC
351 TCGCGCATGGGATCACCTCGCCGGGATGAGTAAACCCTGCCCTGGCGCAG
    GGAGGTTCTCGGGCGGGGCCGACAGGGGCAGGCGCCAGGGAAGGCCAGCA
451 CCCCTGTAACAAGACGACTGTCCCCGCCCACCACTCGGGCCCCCACGCGT
    GCAGCCCTCTTTCATCTCTTGGTCCTCCTTTCTTTCTTTTCATACATGTT
551 ACAGCCACTTCCAAGGAAAGCCTGGATTGCAAGAGCTCTGGGAACCGGAG
    ACTTCAGAGAAGAGGGCTTTGAATGGGGAGTGGGGGAGGTGGTGCACAGG
651 ACCTGCAAGACGCTGGGAGGGGTGATCGGCACCAAGGGCACTTTGGGAGG
    ACCTGCCTAGGACGTGGACTTCCCCGAAGACAGGATCGCAAGGAGAGACA
751 GCTGGATCCTGTCCGCGGCCAAGGTGCCTGGCTCAGGAAACCAGCGGAGC
    GCGCTTGGC
```

Keys:

Regulatory elements:
Green: TATAA box
TSS

Transcription factors binding sites:

ERG binding sites
KLF2 binding site
Sp1/KLF binding sites

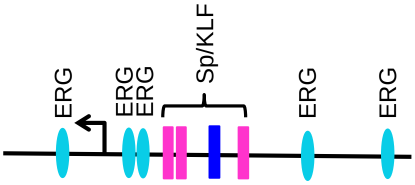

ChIP-qPCR Primers spanning R2
ChIP-qPCR Primers spanning R1

Supplementary Figure 5: Analysis of thrombomodulin locus. (**a**) Thrombomodulin (*TM*) whole locus. ENCODE sequence conservation between 100 vertebrates and ERG ChIP-seq data are shown across this region. ENCODE ChIP-seq data profiles for H3K4Me3, H3K27Ac and RNA Pol II indicate open chromatin and active promoter region. Dashed green box highlights promoter region presented in Figure 4a. (**b**) ERG binding motif sequence used for the analysis of the ERG binding profile on TM promoter was obtained from JASPAR (2018) database (matrix ID: MA0474.2). (**c**) Sequence of ERG binding peak (identified by ChIP-seq) on the TM promoter: -559bp;+215bp around transcription start site (TSS). Sequence was annotated for ERG (light/dark blue) putative DNA binding sites, for known KLF2 (red) and Sp1/KLFs binding sites (pink). Binding sites are also depicted in the right cartoon. TATAA box (green) and Predicted TSS (light grey) obtained from SwitchGear TSS track from ENCODE Project are indicated. Location of ChIP-qPCR primers spanning regions R1 (green) and R2 (yellow) are indicated. Arrow shows direction of transcription.

Sequence of TM promoter luciferase construct (1.078kB)

```
>hg19_dna range=chr20:23030315-23031391 5'pad=0
3'pad=0 strand=+ repeat Masking=none

     TCGGCCAGGGCTCGAGTTTATAAGTGCCCGGCCCTCCCTCCCTGGACGTT
51   CGGGAAAAGGAAGGAAGTGCCTGGTGGGAAGGGCTGATGCCGCATACTCG
     GATTGCTGGGTTCTCTGGCCGCCCTTGCGCCCGCCCTCGCGCATGGGATC
151  ACCTCGCCGGGATGAGTAAACCCTGCCCTGGCGCAGGGAGGTTCTCGGGC
     GGGGCCGACAGGGGCAGGCGCCAGGGAAGGCCAGCACCCCTGTAACAAGA
251  CGACTGTCCCCGCCCACCACTCGGGCCCCCACGCGTGCAGCCCTCTTTCA
     TCTCTTGGTCCTCCTTTCTTTCTTTTCATACATGTTACAGCCACTTCCAA
351  GGAAAGCCTGGATTGCAAGAGCTCTGGGAACCGGAGACTTCAGAGAAGAG
     GGCTTTGAATGGGGAGTGGGGGAGGTGGTGCACAGGACCTGCAAGACGCT
451  GGGAGGGGTGATCGGCACCAAGGGCACTTTGGGAGGACCTGCCTAGGACG
     TGGACTTCCCCGAAGACAGGATCGCAAGGAGAGACAGCTGGATCCTGTCC
551  GCGGCCAAGGTGCCTGGCTCAGGAAACCAGCGGAGCGCGCTTGGCCTCAC
     AGGACAGTGGGTGTGGCTGGGGTGACGGGGCAGGGTGGGGAAGACTGGCC
651  TAACACCAGCGCCCTCTGCCCCATGGCTGGCCAGGGACCCGCGAGTCCCT
     GGACACGCACTGGCCAACGCCAGACCCCATCTCATCGGGTGGGGAAGTCG
751  CGGGGACACTGTCAGGGCGCCGAAGTCCGGACCCGGCTCAGAGGCGGTGG
     CAGGTGAATTGCTGCGGCGCCGGGTAGGGGCGGGCGCGTGGGAGCGAGTC
851  AGCCTGGCCAGTTTCGGCCCAGCTTCCGAAGGATGGTGCTTCTTGCACCC
     CAACAGAGTGGCTGGCAACCCCCCAGGGGAGCGCGCAGGATCCCAGCTGA
951  TCCCACCCGGGTCGGCTAAGGAGGTTTCCATTTCGTCCAGAGTCCGAATT
     GATACCCACGTGCATAGAAACGCCACTTGCTCGGCAAAGGGCACTGAAGA
1051 GCCACCGTCCTGTGGATGGGCAGGGTG
```

Keys:
Green: TATAA box
TSS

ERG binding sites (EBS)

Nucleotide mutations:	
Mutant 1: 2 EBS	Mutant 2: 4 EBS mutants in 1 plus:
59 G to C	383 G to C
60 G to C	384 G to C
63 G to C	573 G to C
64 G to C	

Supplementary Figure 6: Mutagenesis of ERG binding sites on TM promoter. Strategy for the mutagenesis of ERG binding sites (EBS) on TM promoter luciferase construct (1.078kB). DNA sequence of TM promoter construct was annotated for EBS (light blue). TATAA box (green) and Predicted TSS (light grey) are indicated. Arrow shows direction of transcription. Mutagenesis of 2 or 4 EBS was achieved by mutating 4 or 7 G nucleotides (red) into C.

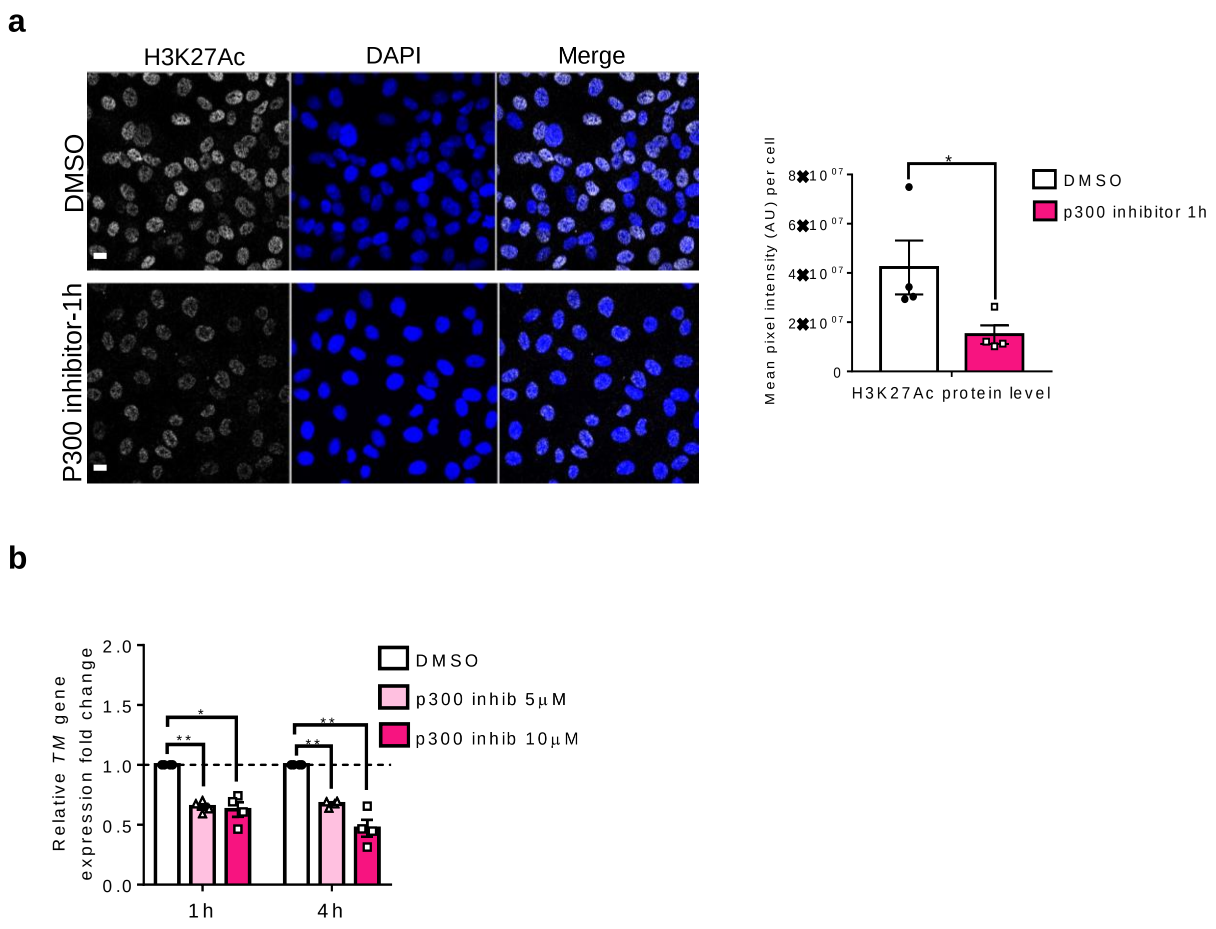

Supplementary Figure 7: P300 inhibition leads to a decreased in H3K27Ac protein level and *TM* mRNA levels in HUVEC (**a**) Representative immunofluorescence image and quantification of H3K27Ac expression (grey) in HUVEC treated with DMSO or p300 inhibitor (10 µM) for 1 hour; nuclei are identified by DAPI (blue). Scale bar 40 µm. Quantification represents the mean pixel intensity for H3K27Ac (arbitrary unit, A.U.) per cell (n=4 independent experiments). Student's t-test. (b) qPCR analysis of *TM* gene expression in HUVEC treated with DMSO, p300 inhibitor 5 µM or 10 µM for 1 hour or 4 hours (n=4 independent experiments). One-Way ANOVA test. All graphical data are mean ±s.e.m., *P < 0.05, **P < 0.01. Source data are provided as a Source Data file.

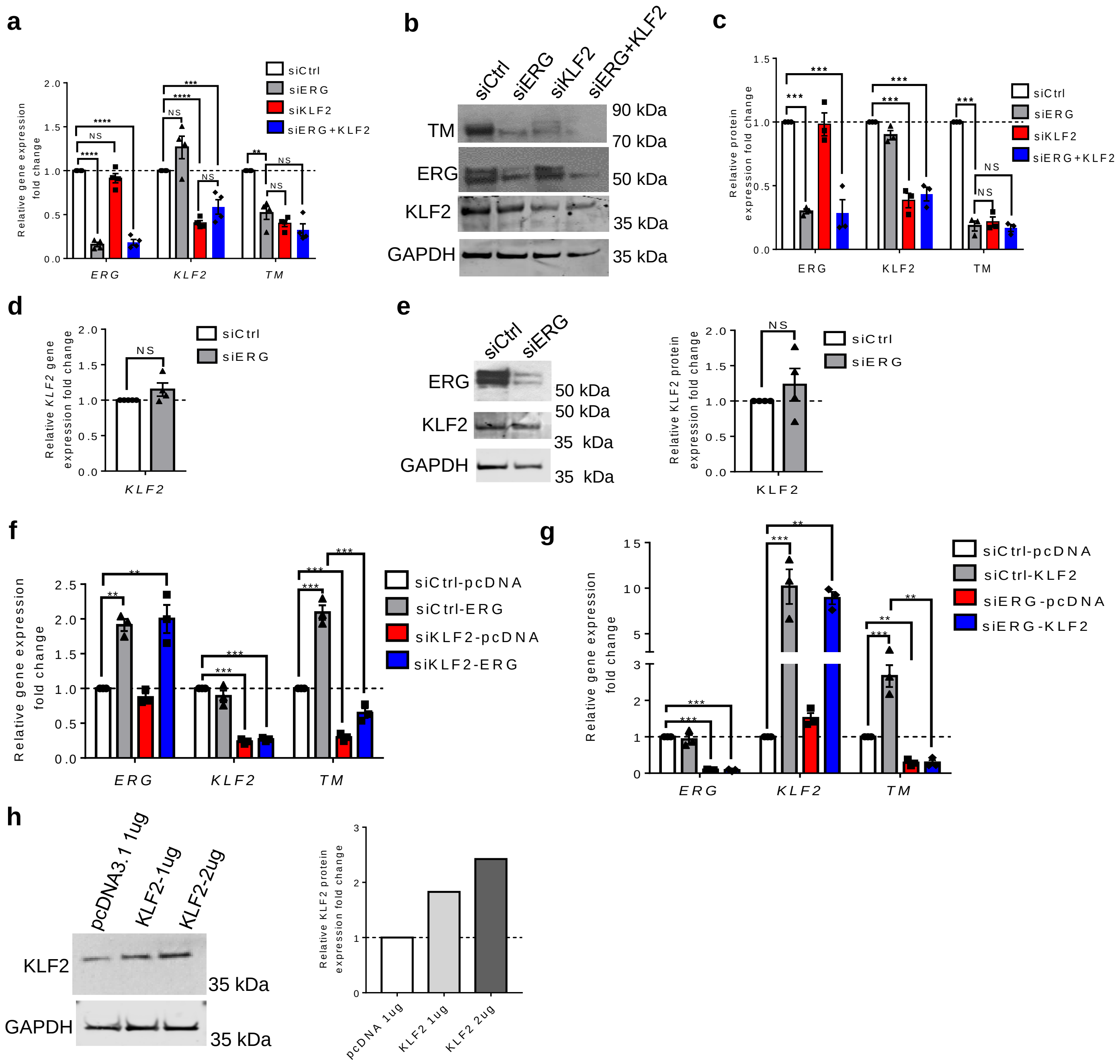

Supplementary Figure 8: ERG cooperates with KLF2 but does not regulate its expression. (**a**) qPCR analysis of *ERG*, *KLF2* and *TM* mRNA expression in siCtrl, siERG, siKLF2 and siERG+KLF2-treated HUVEC after 48 hours (n=4 independent experiments). One-Way ANOVA test. (**b**) Representative immunoblots and (**c**) quantification for ERG, KLF2 and TM protein levels in siCtrl, siERG, siKLF2 and siERG+KLF2-treated HUVEC after 48 hours (n=4 independent experiments). One-Way ANOVA test. (**d**) qPCR and (**e**) immunoblotting analysis of KLF2 levels in siCtrl and siERG-treated HUVEC after 48 hours (n=4 independent experiments). Student's t-test. (**f**) qPCR analysis of *ERG*, *KLF2* and *TM* gene expression following transfection of HUVEC with control or KLF2 siRNA for 48h and transfection with control pcDNA or ERG-myc plasmid (notes ERG) for 24h (n=3 independent experiments). One-Way ANOVA test. (**g**) qPCR analysis of *ERG*, *KLF2* and *TM* gene expression following transfection of HUVEC with control or ERG siRNA for 48h and transfection with control pcDNA or KLF2 plasmid (noted KLF2) for 24h (n=3 independent experiments). One-Way ANOVA test. (**h**) Representative immunoblot and quantification for KLF2 protein level following transfection of HUVEC with control pcDNA or KLF2 plasmid for 24h. All graphical data (except data presented in **h**) are mean ±s.e.m., NS: Not Significant, **P<0.01, ***P<0.001, ****P<0.0001. Source data are provided as a Source Data file.

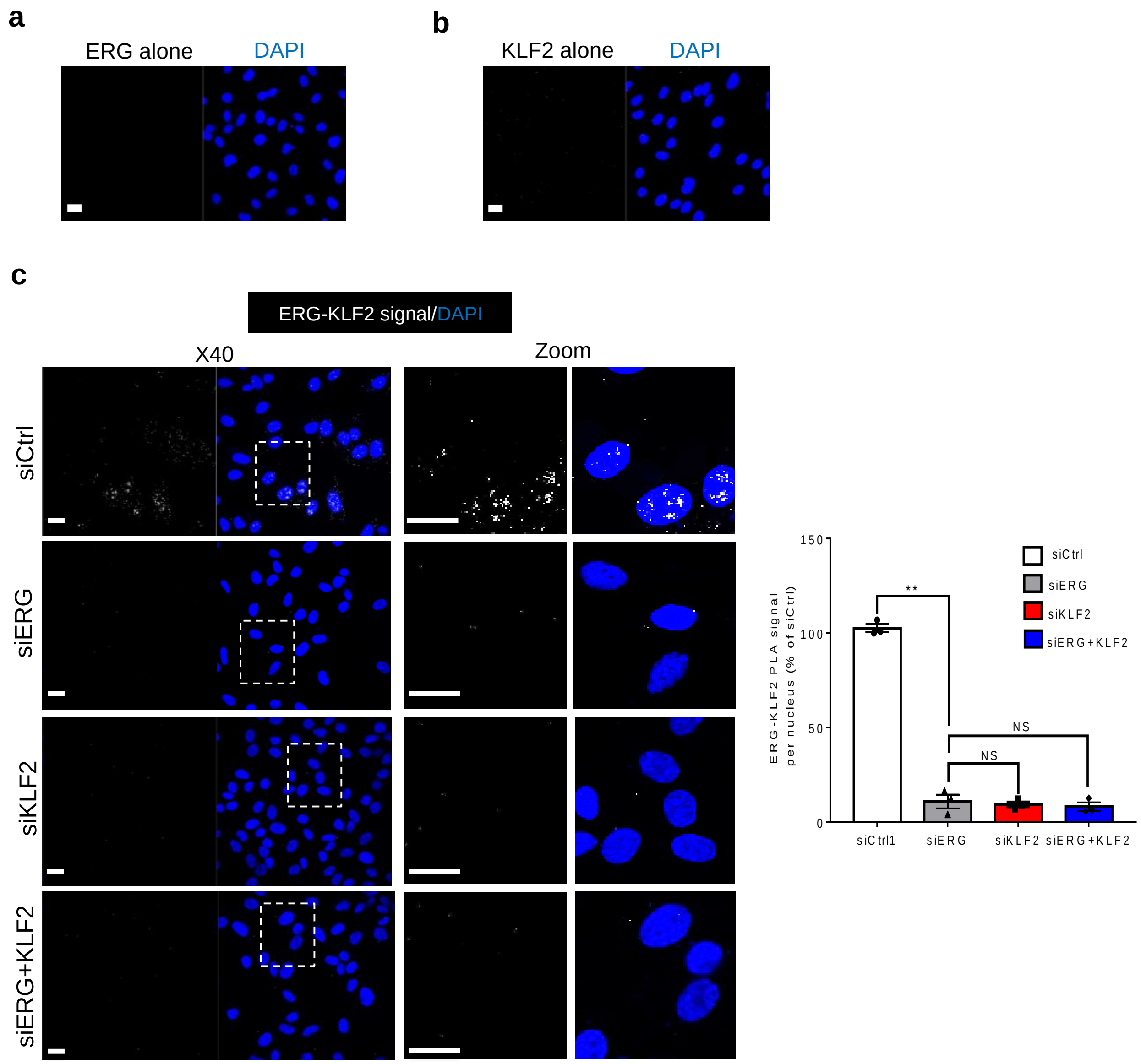

Supplementary Figure 9: Specificity of ERG-KLF2 PLA signal. (**a-c**) Specificity of (**a**) ERG antibody and (**b**) KLF2 antibody used for PLA assay and background signal was established by assessing each primary antibody alone; nuclei are identified by DAPI (blue). Scale bar 20 µm. (**c**) Specificity of ERG and KLF2 PLA signal was also assessed in siCtrl, siERG, siKLF2 and siERG+KLF2-treated HUVEC after 48 hours; nuclei are identified by DAPI (blue). Scale bar 40 µm. Quantification represents ERG-KLF2 signal per nucleus expressed as percentage of siCtrl-treated HUVEC (n=3 images per condition, n=1 experiment). Graphical data are mean ±s.e.m., NS: Not Significant, **P<0.01, One-Way ANOVA test. Source data are provided as a Source Data file.

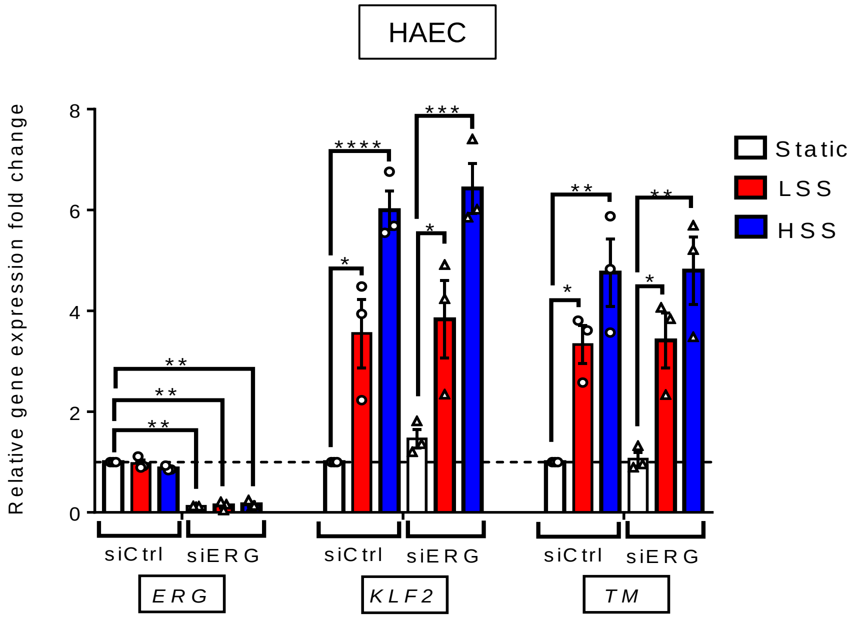

Supplementary Figure 10: ERG does not control the expression of thrombomodulin in human aortic endothelial cells. qPCR analysis of *ERG*, *KLF2* and *TM* mRNA expression in siCtrl or siERG-deficient HAEC under static conditions or after 24 hours under LSS or HSS (n=3 independent experiments) conditions. Graphical data are mean ±s.e.m., *P<0.05, **P<0.01, ***P<0.001, One-Way Anova test. Source data are provided as a Source Data file.

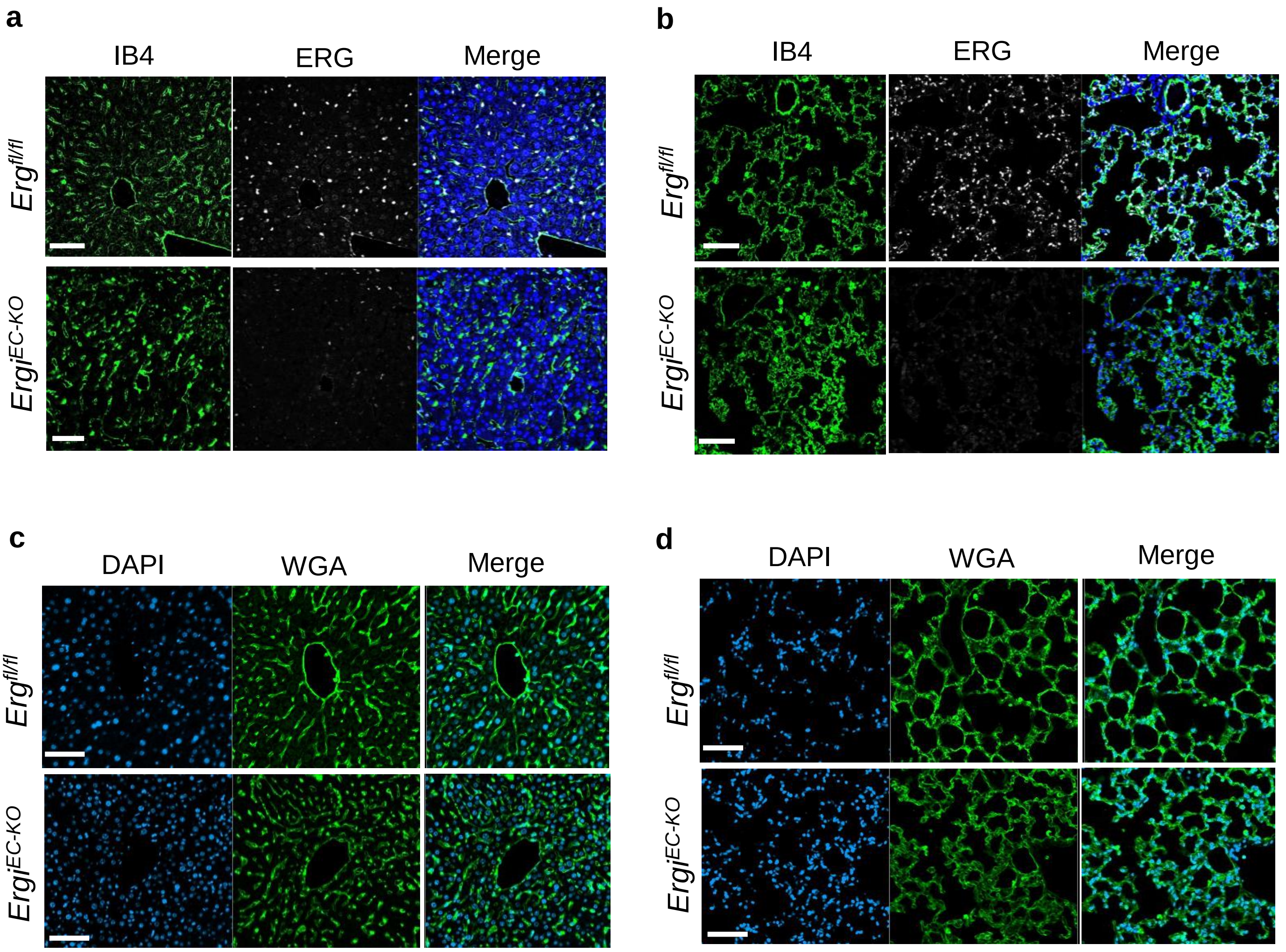

Supplementary Figure 11: Confirmation of ERG knockdown in endothelial cells in mouse liver and lung. (**a-b**) Confirmation of ERG deletion was assessed by immunofluorescence in (**a**) liver and (**b**) lung sections from adult control (*Erg*$^{fl/fl}$) and ERG-deficient (*Erg*$^{iEC\text{-}KO}$) mice 30 days after tamoxifen injection. ERG is shown in grey; sections are co-stained for Isolectin B4 (IB4) (green) to visualize blood vessels and nuclei are identified by DAPI (blue). Scale bar 50 µm. (**c-d**) Representative image of immunofluorescence of wheat germ agglutinin (WGA) expression (green) to visualize tissue architecture in (**c**) liver and (**d**) lung sections from *Erg*$^{fl/fl}$ and *Erg*$^{iEC\text{-}KO}$ mice 45 days after tamoxifen injection; nuclei are identified by DAPI (blue). Scale bar 50 µm.

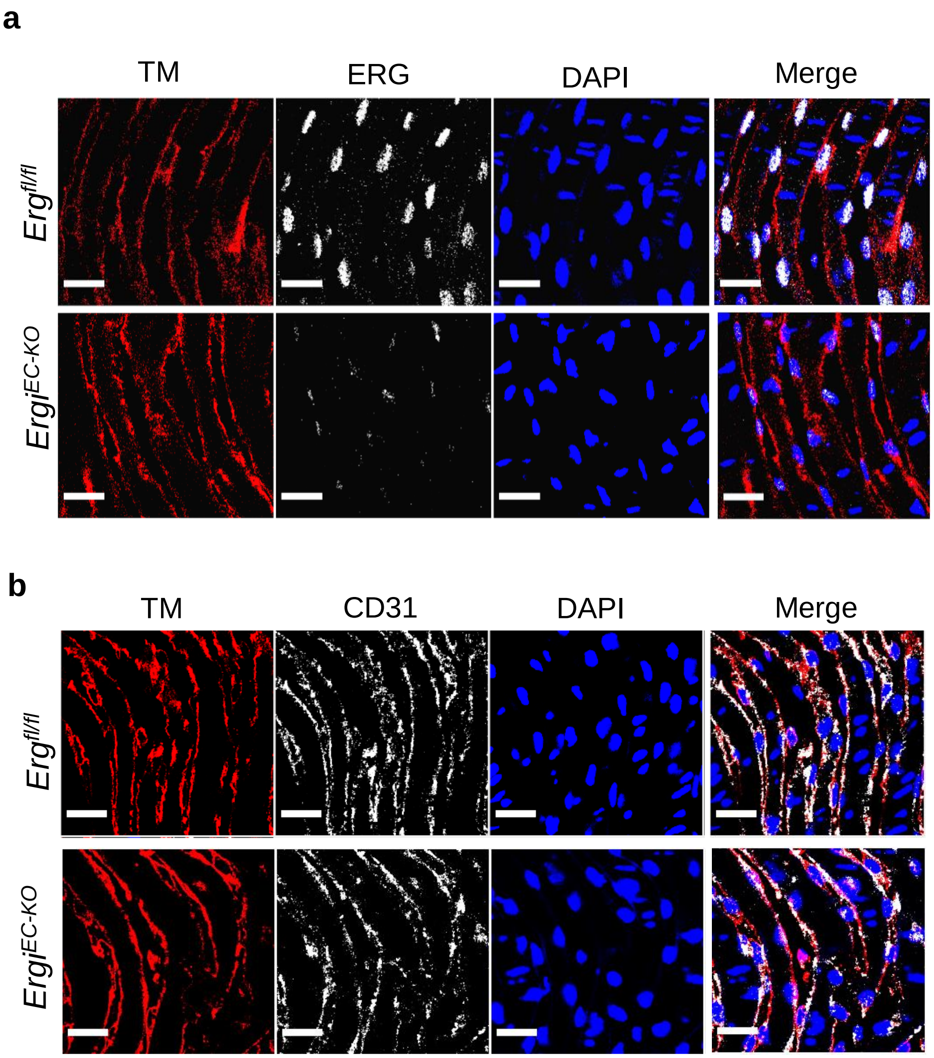

Supplementary Figure 12: ERG does not regulate thrombomodulin expression in aorta *in vivo*. (**a-b**) Representative image of en face staining for TM (red) on the descending aorta (laminar flow region) from adult control (*Erg*$^{fl/fl}$) and ERG-deficient (*Erg*$^{iEC\text{-}KO}$) mice, 30 days post tamoxifen injection. The tissues were co-stained for (**a**) ERG (grey) or (**b**) CD31 (grey) ; nuclei are identified by DAPI (blue). Scale bar 20 µm. Source data are provided as a Source Data file.

Target	Company	Sequence
ERG exon6	Qiagen	5′-CAGATCCTACGCTATGGAGTA-3′
ERG exon7	Invitrogen	5′-ACTCTCCACGGTTAATGCATGCTAG-3′
KLF2	Qiagen	5′-CACCTGGCGCTGCACATGAAA-3′

Supplementary Table 1: Sequences of siRNA used for HUVEC transfection

Antibody (host)	Company	Catalogue number	Application and dilution
CD31 (rabbit)	Abcam	ab28364	IF (mouse tissue, 1/200)
CD41 [MWReg30] (rat)	Abcam	ab33661	IF (mouse tissue, 1/200)
Isolectin B4 (IB4)	Vector	FL-1201	IF (mouse tissue,1/200)
Wheat Germ Agglutinin (WGA)	Thermo Fisher Scientific	W11261	IF (mouse tissue, 1/400)
ERG (mouse)	Santa Cruz	sc-376293	PLA (HUVEC, 1/200)/WB (HUVEC, 1/2000)
ERG (rabbit)	Abcam	ab133264	IF (HUVEC, 1/200)/WB (HUVEC, 1/1000)
ERG (rabbit)	Abcam	ab92513	IF (mouse tissue, 1/200)/WB (mouse tissue, 1/1000)
Fibrinogen (rabbit)	Abcam	ab34269	IF (mouse tissue, 1/200)
GAPDH (mouse)	Millipore	MAB374	WB (HUVEC, mouse tissue, 1/10000)
H3K27Ac (rabbit)	Active Motif	39133	IF (HUVEC, 1/200)
KLF2 (rabbit)	Abcam	ab203591	PLA (HUVEC, 1/100)/WB (HUVEC, 1/500)
Actin, α-smooth muscle-Cy3 (mouse)	Sigma	C6198	IF (mouse tissue, 1/400)
TM (mouse)	Dako	Clone 1009	IF (HUVEC, 1/100)
TM (mouse)	Santa Cruz	sc-13164	WB (HUVEC, 1/1000)
TM (goat)	R&D	AF3894	IF (mouse tissue, 1/200)/WB (mouse tissue, 1/1000)

Supplementary Table 2: List of antibodies used for this study on HUVEC and mouse tissue. IF: Immunofluorescence; WB: Western-Blot; PLA: Proximity ligation assay. Dilution of the antibodies used for each specific application is specified in brackets.

Target	Primers	Oligonucleotide Sequences 5' to 3'
A2M	Forward	GCAGCATAAAGCCCAGTTGC
	Reverse	ATACTGCGGTTTTCCAGAGACT
eNOS	Forward	GTGATGGCGAAGCGAGTGAAGG
	Reverse	ACCACCAGCACCAGCGTCTC
EPCR	Forward	GTCCGGAGCCTCAACTTCAGG
	Reverse	GTGGAACTGGAGCAGGTAGGAC
ERG	Forward	GGAGTGGGCGGTGAAAGA
	Reverse	AAGGATGTCGGCGTTGTAGC
FVIII	Forward	GAAAGTCACAGGAGTAACT
	Reverse	TCCCTGAAAAACCTTTACT
GAPDH	Forward	CAAGGTCATCCATGACAACTTTG
	Reverse	GGGCCATCCACAGTCTTCTG
KLF2	Forward	TTGCAGTGGTAGGGCTTCTC
	Reverse	ACTCACACCTGCAGCTACGC
PAI1	Forward	GCAACGTGGTTTTCTCACCC
	Reverse	GGCCATGCCCTTGTCATCAA
PLAT	Forward	AGAAGCAACCGGGTGGAATA
	Reverse	GGCTCGCTGCAACTTTTGAC
PLAUR	Forward	GAAGGGAAGTTTGTGGCGGA
	Reverse	CAAGAGGCTGGGACGCA
PTGIS	Forward	CTGTGCTTGATAGCGTGCTG
	Reverse	GTCGCAGGTTGAATTCTCGC
PTGS2	Forward	GGCCATGGGGTGGACTTTAA
	Reverse	ACCGTAGATGCTCAGGGACT
TF	Forward	AGTTCAGGAAAGAAAACAGCCA
	Reverse	CGGTTAACTGTTCGGGAGGG
TM	Forward	ACGACTGCTTCGCGCTCTACCC
	Reverse	CACCGAGGAGCGCACTGTCATTA
THBS1	Forward	CAGGAGCAACCTCTACTCCG
	Reverse	CAGCAGGGATCCTGTGTGTA

Supplementary Table 3: List of human oligonucleotides used for qPCR

Target	Primers	Oligonucleotide Sequences 5’ to 3’
18S	Forward	GGACAGGATTGACAGATTGATAG
	Reverse	CTCGTTCGTTATCGGAATTAA
eNOS	Forward	CATCTTCAGCCCCAAACGGA
	Reverse	AGCGGATTGTAGCCTGGAAC
ERG	Forward	CCGGATACTGTGGGGATGA
	Reverse	TCTGCGCTCATTTGTGGTCA
FVIII	Forward	CTTCACCTCCAGGGAAGGACTA
	Reverse	TCCACTTGCAACCATTGTTTTG
PAI1	Forward	AGGATCGAGGTAAACGAGAGC
	Reverse	GCGGGCTGAGATGACAAA
TF	Forward	AGGATGTGACCTGGGCCTAT
	Reverse	GGCTGTCCAAGGTTTGTGTC
TFPI	Forward	CTGGACTCTGCCGAGGTTAC
	Reverse	AGGGGAGTGGACTGGATTCT
TM	Forward	GAAACTTCCCTGGCTCCTATGA
	Reverse	GTCTTTGCTAATCTGACCAGCAA
VWF	Forward	CCGTCTTCAGTAGCTGGCAT
	Reverse	GTGTAAACGGGCATCTCCTC

Supplementary Table 4: List of mouse oligonucleotides used for qPCR

Target	Primers	Oligonucleotide Sequences 5’ to 3’	Application
TM WT Promoter	Forward	ACGTGCTAGCATCCACAGGACGGTGGCTC	PCR amplification of TM Wild Type promoter for reporter construct
	Reverse	ACGTAAGCTTGCTCGAGTTTATAAGTGCCCG	
TM mutant 1 Promoter	Forward	CCTCCCTGGACGTTCGGGAAAACCAACCAAGTGCCTGGTG	Mutagenic primers used for mutation of 2 EBS on TM Wild Type promoter
	Reverse	CACCAGGCACTTGGTTGGTTTTCCCGAACGTCCAGGGAGG	
TM mutant 2a Promoter	Forward	CAAGAGCTCTGGGAACCCCAGACTTCAGAGAAGAGG	Mutagenic primers used for mutation of 1 EBS on TM Wild Type promoter
	Reverse	CCTCTTCTCTGAAGTCTGGGGTTCCCAGAGCTCTTG	
TM mutant 2b Promoter	Forward	GCTCCGCTGGTTTGCTGAGCCAGGCAC	Mutagenic primers used for mutation of 1 EBS on TM Wild Type promoter
	Reverse	GTGCCTGGCTCAGCAAACCAGCGGAGC	
TM PromR1	Forward	CCTCGCCGGGATGAGTAAAC	ChIP-qPCR
	Reverse	CGGGGACAGTCGTCTTGTTA	
TM PromR2	Forward	ACCCCAAGCATGTTACCCAG	ChIP-qPCR
	Reverse	GATCCGCATGTCAGAGGCTG	
TM neg con	Forward	GTGCGCCTTTTCAGAGTGTG	ChIP-qPCR
	Reverse	ACTCTGGCAGGGGAGAAAGA	

Supplementary Table 5: List of human oligonucleotides used for thrombomodulin promoter amplification, mutagenesis and ChIP-qPCR. EBS: ERG binding sites.
